# Advancing Ultrasonic Imaging and Sensing for Automated Nondestructive Testing

**DOI:** 10.3390/s26133993

**Published:** 2026-06-24

**Authors:** Zhenhao Liang, Zhixuan Chang, Siyu Liu, Zesheng Zheng, Haoran Jin

**Affiliations:** 1 The State Key Laboratory of Fluid Power and Mechatronic Systems, College of Mechanical Engineering, Zhejiang University, Hangzhou 310027, China; 12525106@zju.edu.cn (Z.L.); chang_zhixuan@foxmail.com (Z.C.); 2 School of Physics, Nanjing University of Science and Technology, Nanjing 210094, China; 3 Institute of Microelectronics, Agency for Science, Technology and Research, Singapore 138634, Singapore

## 1. Introduction

Ultrasonic Non-Destructive Testing (NDT) technology [1] is advantageous due to its strong material penetration, high sensitivity to internal defects, and operational safety, and plays a critical role in industrial weld inspection, pipeline defect monitoring, structural health monitoring, and biomedical imaging [2,3,4]. Over the past decade, significant progress has been made in ultrasonic NDT. The transition from single-element transducers to phased-array transducers [5] has enabled flexible beamforming and electronic scanning. The introduction of Full Matrix Capture (FMC) and Total Focusing Method (TFM) [6] has brought near-optical resolution to acoustic imaging. More recently, the integration of automation and artificial intelligence has further expanded the capabilities of ultrasonic NDT [7], enabling more efficient, consistent, and sophisticated inspections in environments that are difficult or hazardous for human operators. These developments have extended the application of ultrasonic NDT from initial laboratory and offline testing to online monitoring of critical infrastructure, pipelines, pressure vessels, and aerospace components [8]. However, the involvement of artificial intelligence and automation systems brings greater technical challenges to ultrasonic imaging in terms of computational efficiency, imaging resolution, signal-to-noise ratio, material adaptability, and sensor technology.

This Special Issue, “Ultrasound Imaging and Sensing for Nondestructive Testing”, aims to advance this field by bringing together recent research at the intersection of ultrasonic imaging, sensing, signal processing, and automated inspection systems. Particular attention is given to robotic-assisted and automated ultrasonic imaging systems, theories and algorithms for intelligent testing, advanced ultrasonic sensing technologies, and practical NDT applications in complex industrial settings. The collected papers reflect the ongoing transformation of ultrasonic NDT from conventional signal acquisition and offline processing toward integrated, automated, and intelligent inspection.

## 2. Overview of Contributions

This Special Issue brings together ten accepted papers (nine articles and one review) covering high-performance imaging algorithms, coded excitation, deep learning-based signal processing, micro ultrasonic sensors, and adaptive TFM for anisotropic materials. Together, they provide a comprehensive snapshot of current advances in ultrasonic imaging for next-generation NDT.

These contributions are outlined in more detail below.

Guided wave testing is particularly attractive for large-area inspection because guided waves can propagate over long distances while carrying information about structural defects. However, improving spatial resolution and achieving reliable early-stage damage detection remain two central challenges. The first two papers in this Special Issue address these issues from the perspectives of guided wave mode selection and nonlinear ultrasonic response, respectively.

Contribution 1 addresses the problem of conventional A0 and S0 guided waves having long wavelengths and insufficient resolution for shallow, small-sized corrosion defects. It proposes using the SH1 guided wave mode combined with full waveform inversion (FWI) tomography. Through finite element simulations and experiments, the imaging accuracy of the A0, S0, and SH1 modes for regular and irregular defects was compared. The results show that due to its shorter wavelength (about 16 mm), the SH1 wave is significantly superior to the A0 and S0 modes in reconstructing defect shape, edge profile, and depth, achieving the lowest global relative error (about 0.23–0.37%). This novel guided wave mode demonstrates great potential for improving the imaging accuracy of corrosion defects.

Contribution 2 theoretically and numerically investigates the generation and accumulation characteristics of the zero-frequency component (ZFC) and the second harmonic component (SHC) generated during the propagation of nonlinear Lamb waves in a symmetric undulated plate with periodically varying thickness. It is found that the accumulation of the ZFC is not affected by geometric undulation and can grow linearly with propagation distance, whereas the SHC is limited by phase mismatch and has a finite accumulation distance. This finding indicates that the ZFC-based inspection method is more suitable for early-stage damage assessment in non-uniform thickness plates than the conventional second harmonic method, providing a new physical parameter and more relaxed engineering applicability for guided wave testing.

In the field of ultrasonic imaging, the Total Focusing Method (TFM) has become an important tool for industrial non-destructive testing due to its superior focusing capability. However, there remains room for improvement in terms of computational efficiency, resolution limits, and adaptability to complex materials. The four papers in this Special Issue advance ultrasonic imaging technology from the three perspectives of efficiency, resolution, and material adaptability, laying the foundation for real-time, high-resolution, computationally efficient industrial ultrasonic imaging.

Contribution 3 focuses on the low computational efficiency of conventional time-domain TFM caused by the use of oblique-incidence wedges in weld inspection. It proposes a frequency-domain full-matrix imaging algorithm based on Chebyshev polynomials of the second kind, named the extended direct Chebyshev Fourier (EDCF) method. By directly expanding the Fourier extrapolator to handle lateral sound velocity variations, the method uses a standard extrapolator in regions of uniform sound velocity for efficiency, and a high-order Chebyshev expansion in oblique-incidence regions to ensure accuracy. Over a wide angle range of 0° to 45°, the method maintains imaging quality comparable to TFM while reducing computation time by up to a factor of eight. This method is suitable for fast, high-precision online weld inspection.

Contribution 4 targets the insufficient longitudinal and lateral resolution of the time-of-flight diffraction (TOFD) technique caused by limited transducer bandwidth and acoustic diffraction effects. It proposes an enhanced imaging method that combines time-domain sparse deconvolution (using orthogonal matching pursuit, OMP) with the frequency-domain synthetic aperture focusing technique (F-SAFT). Simulation and pipeline girth weld experimental results demonstrate that the method can clearly delineate defect shape details, improve lateral resolution by about 35%, reduce the average localization error for defect length estimation from 2.2 mm (raw data) to 0.4 mm, and achieve an overall computation time much shorter than the data acquisition time, showing potential for online inspection.

Contribution 5 addresses the problems of uneven energy distribution and high structural noise in the TFM imaging of coarse-grained, elastically anisotropic materials such as austenitic stainless steel welds. It proposes an imaging method that integrates material-property-based acoustic field correction with a vector coherence factor (VCF), named C-VCF-TFM (acoustic field correction vector-coherent total focusing method). Experiments on a nuclear-grade stainless steel nozzle weld specimen containing multiple side-drilled holes proved that, compared with conventional TFM, the method increases the detection signal-to-noise ratio by 2.34–10.95 dB and significantly reduces depth positioning errors, thereby markedly improving defect detectability in coarse-grained material welds.

Contribution 6 addresses the difficulty of defect localization caused by the dependence of sound velocity on crystal orientation in anisotropic materials such as single-crystal turbine blades. It proposes an adapted total focusing method that uses only the full matrix capture (FMC) data itself, avoiding reliance on auxiliary characterization tools such as electron backscatter diffraction (EBSD). Experimental results show that the method improves defect localization accuracy by 61% and increases the signal-to-noise ratio by 5 dB, providing a new technical pathway for efficient, high-precision non-destructive testing of anisotropic materials.

In ultrasonic testing, signal quality and information extraction capability directly affect the reliability of inspection results. From the perspectives of signal processing and intelligent algorithms, the three articles in this Special Issue present effective research approaches addressing the echo interference problem in through-metal ultrasonic communication, the inherent trade-off between signal-to-noise ratio and resolution, and the evaluation of generalizability of deep learning models. Together, these three articles highlight the central role of intelligent signal processing and machine learning in enhancing the performance of ultrasonic testing systems.

Contribution 7 solves the problem of severe echoes (multipath effect) caused by acoustic impedance mismatch in through-metal ultrasonic communication, which leads to inter-symbol interference (ISI). It introduces a dual-path recurrent neural network (DPRNN) for echo cancelation. By segmenting the long-sequence ultrasonic signal into overlapping short blocks and performing intra-block and inter-block RNN modeling, respectively, the method effectively overcomes the vanishing gradient problem of traditional RNNs in long-sequence processing and achieves high-fidelity image transmission.

Contribution 8 systematically reviews the principles, methods, and engineering applications of coded excitation (CE) technology in ultrasonic testing. It details common coding formats such as linear frequency modulation (LFM), Barker codes, Golay codes, polyphase codes (P3, P4), M-sequences, and Gold sequences, along with their pulse compression optimization strategies. The review also discusses the potential of spatial coding (e.g., Hadamard coding) and the combination of spatial and temporal coding. It points out that while coded excitation can effectively decouple the conflict between signal-to-noise ratio and resolution, it still faces practical engineering challenges such as near-field blind zones and transducer heating.

Contribution 9 addresses the difficulty of acoustic time-of-flight (ToF) measurement caused by the overlapping of bulk waves and guided waves in closed containers, and systematically evaluates the generalizability of ToF prediction methods based on convolutional neural networks (CNNs). Using finite element simulations to generate data for cylindrical containers with varying dimensions, wall thicknesses, and sound speeds, the study analyzes the impact of training dataset size, training/testing distribution discrepancy (small-diameter vs. large-diameter), variations in medium sound speed, and data augmentation (noise addition) on model generalizability. This research provides important guidance for the reliable deployment of CNNs in non-destructive evaluation.

The performance of ultrasonic testing depends not only on algorithms but also on the sensitivity and size of the sensors themselves. Contribution 10 designs and validates a miniature ultrasonic sensor based on a multimode microfiber interferometer by exploiting strong evanescent field effects, aiming to overcome the long-standing bottleneck of conventional piezoelectric sensors in balancing sensitivity and size. Experimental results show that the sensor achieves an ultrasound detection limit of approximately 540 Pa, a bandwidth of about 5 MHz, and a sensitivity about five orders of magnitude higher than that of a standard single-mode fiber sensor. Moreover, it achieves an approximately tenfold improvement in resolution in photoacoustic imaging tests, demonstrating great potential for imaging fine-scale structures.

## 3. Conclusions

The ten papers collected in this Special Issue demonstrate recent progress in ultrasonic imaging and sensing for automated and intelligent NDT. These contributions cover guided wave mode selection, nonlinear ultrasonic testing, high-efficiency imaging algorithms, adaptive TFM, coded excitation, deep learning-assisted signal processing, and miniature ultrasonic sensors. Together, they provide new approaches for achieving higher imaging resolution, improved signal-to-noise ratio, enhanced material adaptability, more accurate defect localization, and better potential for real-time deployment. Despite these achievements, prominent challenges remain in constructing large-scale, standardized ultrasonic defect datasets and evaluation benchmarks for deep learning, realizing multi-parameter ultrasonic information fusion, and enabling the efficient and robust practical deployment of offline algorithms into online systems. In particular, the high-speed real-time processing of massive data, the lightweighting of model structures, and the capability of algorithms to adapt to dynamic inspection environments and variable defect morphologies remain pressing challenges that urgently need to be overcome.

We hope that this Special Issue will inspire further research and innovation in ultrasonic imaging and sensing for NDT, and will help bridge the gap between laboratory research and industrial applications. Finally, we sincerely thank all authors and reviewers, as well as the Editorial team, for their valuable contributions and dedicated efforts. We look forward to the continued development of this rapidly evolving field.
